# Increase in Strength After Multimodal Pain Management Concept in Patients with Cervical Radiculopathy—A Non-Randomized, Uncontrolled Clinical Trial

**DOI:** 10.3390/medicina60121961

**Published:** 2024-11-28

**Authors:** Gerd Zirkl, Jens Schaumburger, Matthias Gehentges, Moritz Kaiser

**Affiliations:** 1 Dr. Kaiser & Kolleginnen MVZ GmbH, Puricellistraße 34, 93049 Regensburg, Germany; 2 Department of Orthopedic Surgery, Regensburg University, Medical Center, 93077 Bad Abbach, Germany; 3 Orthopädie Mühleninsel, Mühlenstrasse 1–3, 84028 Landshut, Germany; 4 Department of Pediatrics and Adolescent Medicine, St. Marien Hospital Amberg, Mariahilfbergweg 7, 92224 Amberg, Germany

**Keywords:** cervical radiculopathy, multimodal pain management, hand-held dynamometer, muscle strength

## Abstract

*Background and Objective*: Although multimodal pain therapy (MPT) is widely used in pain management for chronic cervical radiculopathy, its effect on increasing muscle strength in patients with cervical radiculopathy is not well documented. This study aimed to evaluate the impact of a structured multimodal pain management program on muscle strength in these patients, using objective strength measurements as indicators of therapeutic success. *Materials and Methods*: This non-randomized, uncontrolled, prospective clinical study initially included 35 patients, but 10 were excluded due to incomplete data, resulting in a final sample of 25 patients (14 women and 11 men, aged 42 to 84 years) with cervical radiculopathy who underwent a multimodal pain management program at a specialized orthopedic clinic. Muscle strength was measured at admission and discharge using a hand-held dynamometer. Pain levels were assessed with the Numeric Rating Scale. An uncontrolled study design was chosen for ethical reasons, as it was deemed inappropriate to form a control group that would be deprived of necessary anti-inflammatory or analgesic medications. *Result*: Significant improvements were observed in overall muscle strength, with an 11% increase from 114.78 kg to 127.41 kg (*p* = 0.003). The strongest increase in strength was observed in the proximal cervical muscle groups, with a notable 22.9% (*p* < 0.001)improvement in the muscles involved in cervical inclination. However, no significant strength gains were detected in the peripheral muscle groups of the upper arm. Pain scores on the NRS decreased by 54.2% (*p* < 0.001). *Conclusions*: This study demonstrates that a multimodal pain management approach leads to significant improvements in muscle strength and pain reduction in patients with cervical radiculopathy. The increase in cervical muscle strength is closely associated with pain relief and improved functional outcomes, highlighting the value of conservative pain management strategies for these patients.

## 1. Introduction

Cervical radiculopathy is characterized by pain radiating to one or both upper limbs, often accompanied by neck pain, resulting from the compression or irritation of cervical nerve roots. This condition is frequently associated with motor, sensory, or reflex deficits, and is most prevalent in individuals aged 50 to 54. The primary etiology of cervical radiculopathy is cervical spine degeneration, which progresses with age and mechanical stress [1].

Neck pain itself is a widespread health issue globally. The prevalence rate among individuals aged 40 and older in developed countries is nearly 20% [2]. According to the latest Global Burden of Disease data, neck pain is the fourth leading cause of years lived with disability, following only back pain, major depressive disorder, and arthralgias [3]. Globally, in 2020, approximately 203 million people were affected by neck pain. By 2050, the estimated global number of neck pain cases is projected to reach 269 million, representing an increase of 32.5% (23.9–42.3) from 2020 to 2050. Population growth is considered the primary contributing factor, followed by population aging [3]. While acute neck pain typically has a favorable prognosis, a substantial proportion of patients (37–47%) report persistent pain one year post-onset [4].

Multiple risk factors contribute to the development of neck pain. Unmodifiable risk factors, such as female gender and older age, play a role [5,6]. Additionally, psychological factors, including depression, anxiety, and catastrophizing, are linked to increased risk [7,8,9], alongside impaired endogenous pain inhibition [10]. Verwoerd et al. investigated prognostic factors for the development of chronic neck pain following a first episode of idiopathic, non-traumatic neck pain. In a systematic review, they identified ‘age > 40 years’ and ‘concomitant back pain’ as prognostic factors for pain intensity. For the outcome of “perceived non-recovery”, a previous episode of neck pain and accompanying headaches were found to be relevant prognostic factors [11].

Another major risk factor is poor endurance of the cervical spine extensors, which may predispose individuals to chronic neck pain [12,13].

Multimodal pain therapy, a comprehensive treatment strategy integrating physical, psychological, and pharmacological interventions, has been extensively studied for low back pain, and its effectiveness is well documented [14,15]. Emerging evidence suggests that this approach is equally beneficial for neck pain patients. A study involving 365 patients who underwent inpatient multimodal pain therapy showed significant improvements in key outcomes such as return-to-work status, health pain intensity, functional capacity, and therapy satisfaction, indicating that patients with chronic neck pain derive substantial benefits from a multidisciplinary approach [16].

It is estimated that the neck muscles contribute approximately 80% of the stability of the cervical spine, with the osteoligamentous system providing the remaining 20% [17]. This underscores the critical role of muscle strength in cervical spine function, and the potential for dysfunction to be mitigated through strengthening exercises. The importance of strengthening the neck muscles for recovery is well documented [18]. Several studies have shown that increasing the isometric strength of the cervical spine during rehabilitation is strongly associated with pain reduction [19,20]. Additionally, endurance training has demonstrated similarly positive effects [21,22], further emphasizing the importance of both strength and endurance training in managing chronic neck pain.

## 2. Aim of This Study

Numerous clinical studies on multimodal pain therapy for patients with neck and upper extremity pain have primarily used subjective parameters, such as pain reduction, to assess treatment success.

This study primarily focuses on the impact of a structured multimodal pain management program on muscle strength in neighboring muscle groups in patients with cervical radiculopathy, using objective strength measurements as an indicator of treatment efficacy.

## 3. Material and Methods

This non-randomized, non-mixed, prospective clinical study included male and female patients with cervical radiculopathy who were treated in inpatient multimodal pain therapy at the Department of Orthopedic Surgery, Regensburg University Medical Center, Bad Abbach, Germany, between June 2018 and April 2019. Clear indications for multimodal pain therapy arise after at least 6 weeks of unsuccessful outpatient treatment with persistent symptoms, provided there is no absolute indication for surgery.

A total of 35 participants (15 male and 20 female) were initially recruited for the study. Patients were recruited for MPM treatment in the outpatient clinic if they met the specified criteria. Enrollment in the study occurred on the first day of their hospital stay. Of the 35 participants, 25 (11 male and 14 female) completed both the admission and discharge assessments. Ten participants (4 male and 6 female) were excluded due to incomplete data. Eight patients were discharged early, one patient was lost to follow-up for the planned final assessment, and one patient developed severe lumbar pain and received further treatment for this condition. On average, the 25 participants remained hospitalized for 10.48 days (minimum 8 days, maximum 13 days) for the implementation of the MPM.

The study included patients with chronic neck and arm pain, presenting clear signs of radiculopathy and a pain intensity of at least 2 on the Numeric Rating Scale (NRS 0-10). A definitive association of pain symptoms with a specific nerve root and/or muscle weakness due to nerve root involvement was considered indicative of radicular symptoms. The corresponding clinical examination results were supported by MRI imaging.

Exclusion criteria included the presence of myelopathy, recent surgical intervention for complaints in the region of interest, acute or chronic infection, congenital spinal malformations, and spinal tumors.

This prospective study was approved by the local ethics committee (Nr. 18-931-101) and conducted in accordance with the Declaration of Helsinki of 1975. Written informed consent was given by all patients participating in this study, which was voluntary. For ethical reasons, a control group receiving a placebo instead of anti-inflammatory and analgesic medication or cervical nerve root injections was not established.

### 3.1. Measurement

Standardized strength measurements were performed on each patient at admission and prior to discharge using a ‘MicroFet 2’ hand-held dynamometer (Hoggan Health Industries, Inc., 8020 South 1300 West, West Jordan, UT, USA). These measurements were independently conducted for each patient by two examiners trained in the use of the device. The contact point of the dynamometer on the patient`s skin was selected to achieve the maximum possible leverage. To maintain consistency, the location was marked with a pen, and patients were instructed to renew the markings after personal hygiene. This approach ensured the comparability of measurements both before and after treatment, as well as across groups.

Measurements were taken using the ‘break technique’, a specific method for determining the maximum muscle strength of a muscle group. In this technique, the patient pushes against the resistance of the hand-held dynamometer until muscle strength can no longer be maintained. At this point, the resistance is briefly held while the patient exerts maximum force. Compared to the ‘make technique’, where the patient performs a maximum isometric contraction while the examiner holds the dynamometer in a fixed position, the break technique yields higher values. However, both methods provide strength measurements with excellent reliability [23]. The measurement results were recorded in tabular form, expressed in kilograms.

For testing the cervical region, the patient was instructed to stand with their back against a wall to prevent movement away from the device. Reclination was measured with the patient standing sideways to the wall. Contact points were selected to ensure both patient comfort and maximum leverage. The dynamometer was positioned, and the patient was instructed to perform the corresponding movements to execute the break technique (as described above) for neck reclination, inclination, head rotation to the right and left, and flexion and extension of the elbow on both sides.

This study focused on measuring cervical inclination, reclination, and rotation, as these movements are clinically relevant for assessing function in patients with cervical radiculopathy. Since the primary muscles and nerve roots (C2–C4) involved in lateral flexion overlap significantly with those responsible for rotation, its measurement would have provided limited additional value in this context.

The validity and reliability of this pain assessment tool have already been demonstrated in the context of back pain [24]. The NRS in our study was recorded once at the start of therapy during the admission interview and again at the discharge interview upon completion of treatment.

### 3.2. Therapeutic Procedure

The primary goals of multimodal pain therapy are to reduce pain and enhance pain management, as well as to improve physical function and quality of life. Additionally, it aims to teach coping strategies that enable patients to manage their pain more effectively, leading to a lasting improvement in their overall well-being. This treatment approach is based on a combination of various therapeutic methods that complement each other, ensuring comprehensive and personalized pain management. A prerequisite for performing targeted strengthening exercises is that the patient can perform the exercises with minimal pain, which is achieved within the MPM framework through infiltrations with anti-inflammatory and analgesic medications.

Each patient undergoing inpatient multimodal pain therapy received an average of two injections per day as part of cervical spinal nerve root analgesia (CSPA), one in the morning and one in the afternoon. In this procedure, 0.5% mepivacaine was injected into the targeted nerve root using the ‘free hands technique’. Additionally, each patient received a single cervical epidural translaminar steroid injection during their inpatient stay. This procedure involved the injection of 40 mg triamcinolone along with the contrast agent Solutrast 250 in a sterile operating room setting. The intervention was performed under X-ray guidance in two planes using the loss-of-resistance technique. Cervical facet joint infiltrations and occipital nerve infiltrations were also conducted.

In addition to the daily infiltration treatment, patients participated in an intensive exercise program focused on stabilization, isometric strengthening exercises, and coordination training. This was conducted under physiotherapeutic supervision. Regularly conducted group-based aquatic gymnastics complemented the treatment with water-based exercises aimed at improving flexibility, strength, and stability under reduced load. Additionally, individualized equipment training was provided to enhance overall strengthening and support for the cervical spine. Both components were performed under therapeutic supervision. Additionally, progressive muscle relaxation following Jacobson’s method and regular physical therapy procedures were implemented to promote relaxation and muscle toning.

The psychotherapeutic program includes an individual interview, with psychological discussions centered, for example, on individual perspectives and personalized pain management techniques, but is primarily centered on group therapy, focusing more on practical and interactive elements. Group therapy engages participants in discussions that enhance collective awareness among members, with the effectiveness of these components well supported in the literature. A core goal of the program is to provide condition-specific information, promote health and vitality, and reduce psychophysiological arousal triggered by stressors, especially pain. Another key component of multimodal pain management (MPM) is understanding pain perception and learning pain management strategies. This aspect was facilitated through focused discussions with psychologists and structured group training sessions (Table 1).

### 3.3. Statistical Analysis

For the statistical analysis, data were continuously presented as mean values and standard deviation. Group comparisons were conducted using a two-sided *t*-test for the dependent variables. Absolute and relative frequencies were reported for categorical data. Interobserver agreement was evaluated using the intraclass correlation coefficient (ICC). The following values were determined according to Koo TK [25]: An ICC of less than 0.5 indicated poor reliability, between 0.5 and 0.75 was considered moderate, between 0.75 and 0.9 was good, and greater than 0.9 indicated excellent reliability. Differences with a *p*-value of <0.05 were considered statistically significant. The analysis was conducted using IBM SPSS Statistics 25 (SPSS Inc., Chicago, IL, USA).

## 4. Results

### 4.1. Patients

**Figure 1:**
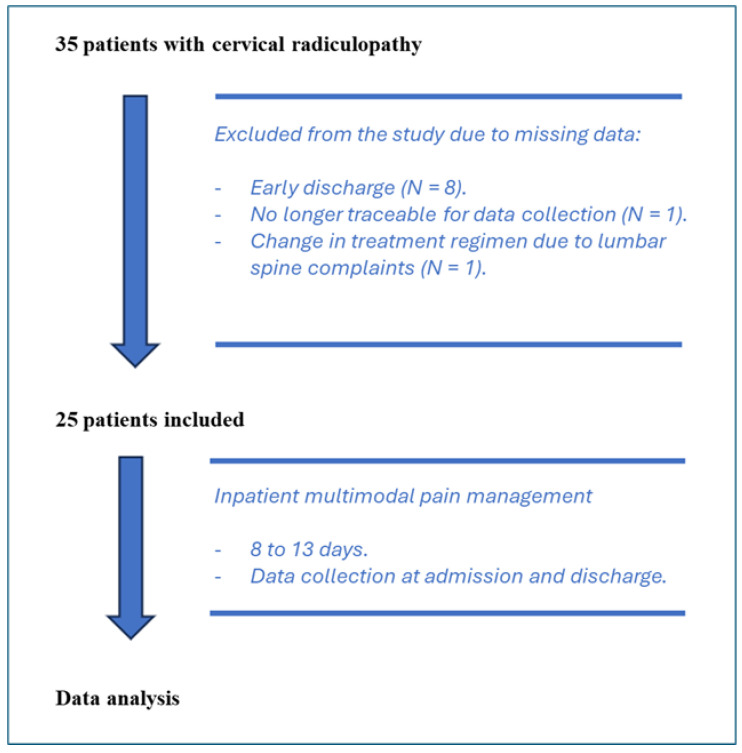
Flowchart of patient inclusion.

A total of 35 participants were initially enrolled in the study. Of these, 25 completed both the admission and discharge evaluations, while 10 were excluded due to incomplete data: 8 patients were discharged early, 1 was lost to follow-up, and 1 developed severe lumbar pain requiring further treatment (Figure 1). The participants included in the study had a mean age of 59.64 years, ranging from 42 to 84 years. The average BMI was 27.4, with a mean height of 171.72 cm and an average weight of 81.02 kg (Table 2). Two participants received continued medication treatment due to a pre-existing diagnosis of depression, one of whom also suffered from an existing anxiety disorder. One participant reported experiencing sleep disturbances and required regular medication as well.

### 4.2. Interrater Reliability

Overall interrater reliability for measurements taken before treatment, on the day of admission, was excellent with an ICC of 0.98. Similarly, interrater reliability for measurements taken after treatment, at discharge, was also excellent with an ICC of 0.97.

### 4.3. Pain Development

**Figure 2:**
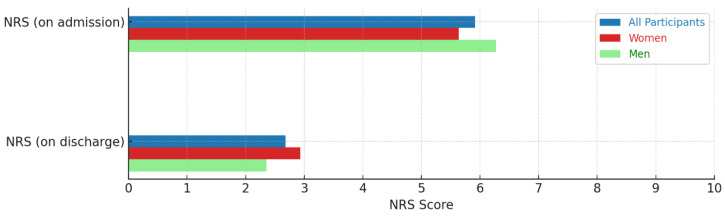
Representation of NRS from admission to discharge.

The NRS was recorded at admission and discharge. Pain levels on the Numeric Rating Scale (NRS) decreased by 54.2%, from 5.9 before treatment to 2.7 after treatment (*p* < 0.001) during the inpatient stay (Figure 2).

### 4.4. Developing Strengths

Overall strength increased significantly by 11%, from 114.78 ± 39.28 kg before treatment to 127.41 ± 42.27 kg after treatment (*p* = 0.003).

Cervical inclination increased significantly by 18.3%, from an average of 10.79 kg on the first day to 12.76 kg at discharge (*p* = 0.005). Reclination also showed a significant increase of 22.9%, from 10.5 kg before treatment to 12.9 kg afterward (*p* < 0.001). Additionally, there was a significant improvement in muscle strength for cervical spine rotation after therapy. On the right side, strength increased by 21.8%, from 10.1 kg to 12.3 kg (*p* < 0.001), while the left side, it showed a significant increase of 18.4%, from 9.8 kg to 11.6 kg (*p* = 0.001).

**Figure 3:**
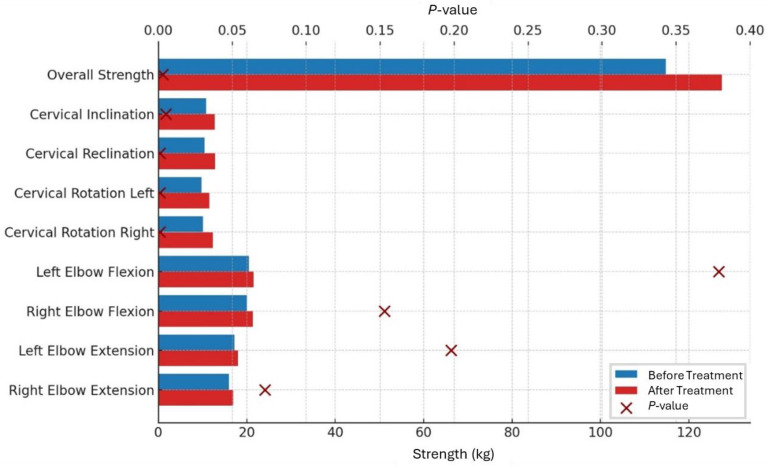
Muscle strength before and after treatment with *p*-value.

**Figure 4:**
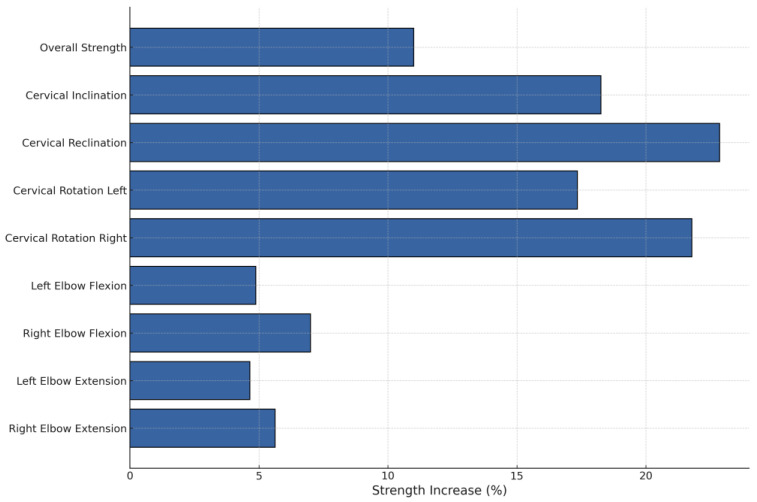
Strength increases in various muscle groups.

Peripheral muscle groups showed an increase in strength, though this was not statistically significant. For right elbow flexion, strength increased by 7%, and for left elbow flexion by 4.9%, from 20.0 kg to 21.4 kg (*p* = 0.153) and from 20.5 kg to 21.5 kg (*p* = 0.379), respectively. Elbow extension strength increased by 5.6% on the right and 4.7% on the left, with absolute values rising from 16.0 kg to 16.9 kg (*p* = 0.072) and from 17.2 kg to 18.0 kg (*p* = 0.198), respectively (Table 3, Figure 3 and Figure 4).

**Figure 5:**
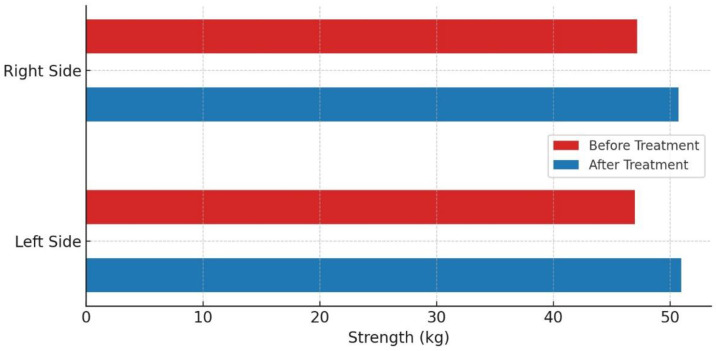
Comparing the strength of the left and right sides before and after treatment.

No significant difference was observed between the left and right sides before and after treatment. Before treatment, the values were 47.0 kg for the left side and 47.2 kg for the right side (*p* = 0.9). After treatment, these values increased to 50.97 kg for the left side and 50.72 kg for the right side (*p* = 0.824) (Figure 5).

## 5. Discussion

To date, no objective data are available on the change in muscle strength in patients undergoing comparable multimodal pain treatment for neck pain, specifically cervical radiculopathy. Most studies in this area have primarily evaluated outcomes like pain reduction, range of motion, and disability but have not extensively focused on quantifying muscle strength improvements.

Therefore, the main purpose of this study was to objectify the effects of multimodal pain treatment on the muscle strength development of the cervical spine and peripheral muscle groups using a hand-held dynamometer.

Overall, comparisons of existing data with the literature do not appear to be clear due to uneven measurement methods.

Compared to low back pain, there is limited clear evidence in the literature regarding the effectiveness of multimodal pain therapy for neck pain. The behavioral therapy component seems to play a critical role in achieving long-term therapeutic effects.

Schonstein et al. report that physical conditioning programs incorporating a cognitive-behavioral approach can significantly reduce the number of sick days in workers with chronic back pain when compared to usual care. Additionally, these programs resulted in improvements in pain relief and a reduction in disability [26].

A study conducted to quantify outcomes based on the evaluation of questionnaires administered 12 months after a three-week multidisciplinary therapy program demonstrated significant improvements in both pain and functional capacity in subjects with pre-existing neck complaints [27].

Similar results were obtained in a randomized, controlled study of 46 women with chronic neck pain, who took part in a specific exercise program. The intervention led to better muscle control and reduced muscular co-contraction, contributing to overall pain relief and improved function [28].

In a prospective cohort study, Weigl et al. investigated prognostic factors associated with improvements in chronic neck pain following a multidisciplinary rehabilitation program. A total of 112 patients were assessed for pain and disability at the beginning of a three-week rehabilitation program, at discharge, and again six months post-program, using the NASS questionnaire. Significant improvements in pain and disability were observed both at discharge and at the six-month follow-up. Key prognostic factors included lower baseline scores for pain and disability, good cervical range of motion, older age, and better mental health status [29].

However, there are also authors who observed no or only minimal effects. Karjalainen et al. investigated in a systematic review of randomized controlled trials the impact of multidisciplinary biopsychosocial rehabilitation on neck and shoulder pain in working-age adults. The study found modest short-term improvements in pain relief and functional outcomes compared to standard care, though long-term benefits were less clear. The approach was particularly effective for individuals with more severe pain and disability [30].

In our study, strength was measured using a hand dynamometer in an upright position. Previous studies have demonstrated that body posture significantly influences isometric force development [31], with greater force generated in the prone position compared to the seated position [32]. A study by Martins et al. investigated neck strength measurements using a handheld dynamometer in both seated and standing positions, examining differences in measurement accuracy between these two positions. The study demonstrated that both positions exhibited similar reliability values, suggesting that both are suitable for measuring isometric neck strength [33].

Ylinen et al. conducted isometric peak force measurements using a hand-held dynamometer on both symptom-free subjects and individuals suffering from chronic neck complaints. The study revealed that the neck pain group exhibited significantly lower neck muscle strength compared to the control group across all tested directions. Specifically, muscle strength was reduced by 29% in extension, 29% in flexion, and 23% in rotation [34]. Therefore, this factor should be considered when planning a rehabilitation program, as it may also serve as an indicator of therapeutic outcomes. It is important to note that lateral flexion and head rotation involve similar neuromuscular structures, particularly the C2-C4 nerve roots and muscles such as the sternocleidomastoid and scalene group. The strength gains observed in rotation during the study likely reflect corresponding improvements in lateral flexion, even though the latter was not directly measured.

It can also be assumed from this perspective that the spinal injections administered as part of multimodal therapy management in our study enhanced the effectiveness of functional training. Studies have shown that epidural injections likely reduce the local inflammatory response, thereby improving functional treatability, which collectively leads to a better overall outcome [35].

For cervical reclination, we observed a significant increase in strength of 22.9%, with values rising from 10.5 kg before treatment to 12.9 kg after the intervention.

Physiotherapy interventions with a multimodal approach have proven effective in improving function and strength [36,37]. However, there is a notable lack of recent studies that objectively measure strength gains in the cervical musculature

Ylinen and Ruuska investigated in an older study the effect of a 3-week rehabilitation program on 56 patients with neck and shoulder pain. The study reported an increase in cervical spine extension strength from 158 N (16.1 kg) to 207 N (21.1 kg), and an increase in flexion strength from 83 N (8.5 kg) to 117 N (11.9 kg). This corresponds to a 30.3% improvement in extension strength and a 40% improvement in flexion strength [38]. This study was primarily focused on isometric strength measurements of the neck muscles. Pain and functional assessments were not the central aspects of this particular study.

Bohannon et al. investigated the increase in neck retraction force in patients referred for physiotherapy, using a similar test setup with a hand-held dynamometer to measure force. The study demonstrated a significant increase in neck retraction force, from 76.5 N (7.8 kg) to 119.5 N (12.2 kg) over the course of treatment, representing a 56.2% increase in strength [39]. An important limitation of this study lays on the fact that it follows neither a standardized protocol nor a consistent time frame. On the other hand, the authors have not included a sufficiently detailed description of their intervention to determine if it follows a standardized protocol or has been performed by using a consistent time frame. Additionally, the measurements were performed by the patients themselves after an initial instruction, which could affect the accuracy and consistency of the results. The study confirmed that dynamometer measurements are both valid and responsive for tracking changes in neck strength during rehabilitation, although variations in patient performance and measurement techniques may influence the results.

A significant increase in cervical spine extension strength, along with improved mobility, was demonstrated after 8 weeks of therapeutic training in a study conducted in 1992 [40]. However, these were primarily patients with neck strain who had not undergone multimodal pain therapy.

In a study with a similar test setup, the research group was able to determine an average cervical retraction strength of 166.7 N (16.99 kg) when examining healthy subjects [41]. The aim of this research was to analyze the reliability and validity of measurements taken with a hand-held dynamometer and to investigate cervical retraction force in the general population. The results demonstrated high reliability, with an Intraclass Correlation Coefficient (ICC) of 0.97, which is comparable to the excellent ICC values observed in our study: 0.98 before treatment and 0.97 after treatment. These findings confirm the consistency and validity of hand-held dynamometer measurements for assessing cervical retraction strength. Other studies that investigated the reliability of dynamometers for measuring neck strength also reported similar results, demonstrating high reliability in assessing cervical muscle strength [42,43], or at least demonstrated moderate to good intra- and interrater reliability [44].

Harries et al. investigated the interrater reliability of a non-instrumented clinical test for assessing neck flexor muscle endurance in individuals with and without neck pain. In asymptomatic subjects, the interrater reliability was found to be moderate, with an ICC of 0.67. For participants with neck pain, the ICC ranged from 0.67 to 0.78, indicating moderate to good reliability [45]. These results suggest that a device-based approach, such as the hand-held dynamometer, may be superior to non-instrumented tests in terms of accuracy and reproducibility.

Even though the strength of the neck flexors is considerably lower than the strength of the neck extensors (1:1.37 for men and 1:1.79 for women) [46] it is important to ensure sufficient strength in the neck flexor muscles when treating neck pain.

A comparative study on isometric strength and endurance performance of cervical flexors and extensors in women with and without neck pain showed that the neck pain group exhibited significantly lower endurance times for the flexors compared to the asymptomatic group. Additionally, both the strength of the flexors and extensors and the ratio of cervicothoracic extension to craniocervical flexion strength were lower in the idiopathic neck pain group [47]. This suggests that strengthening the neck flexor muscles could also have positive effects in the treatment of neck pain, potentially improving overall muscle balance and reducing pain symptoms.

Falla et al. demonstrated that even isolated, specific training of the deep neck flexor muscles can effectively reduce pain and improve muscle activation. In the study, female participants with chronic neck pain underwent a six-week specific training program, which involved performing a craniocervical flexion exercise twice daily throughout the study [48]. A similar result was obtained in a study investigating the effects of isolated deep neck flexor muscle training on pain reduction using self-reported questionnaires [49].

The participants in our study demonstrated a 22.9% increase in cervical extensor muscle strength and an 18.3% increase in cervical flexor muscle strength as part of multimodal pain therapy. The average strength values at admission were 10.5 kg (reclination) and 10.79 kg (inclination), increasing to 12.9 kg and 12.76 kg, respectively, at discharge. Based on these results, it can be inferred that the trained increase in strength positively influenced patient outcomes, contributing to improved functional capacity and pain reduction.

## 6. Limitation

There are several limitations in our study. First, we did not include information regarding the patient’s dominant side. Since the dominant side is often stronger, not accounting for this factor could have led to skewed results. Additionally, force values were measured only at the beginning and end of therapy, so no data are available on the progression of strength development during treatment. A limitation of this study is the lack of measurement for lateral flexion. Including this movement could have allowed for a more comprehensive assessment of cervical muscle strength. However, due to the neuromuscular overlap with rotation, we believe that the movements selected in this study are sufficient to adequately represent the therapeutic effects. We utilized a hand-held dynamometer for freehand techniques, which is more dependent on the motor control of the evaluator compared to fixed measurement devices. This could result in a learning effect for the rater over time, potentially influencing the measured force values. Furthermore, with two different testers involved, factors such as gender, body weight, and the strength of the tester may have been important determinants of both the magnitude and reliability of the measured forces [50,51,52]. This study did not include a control group, which limits the ability to compare the observed outcomes to those receiving a placebo or alternative treatments.

## 7. Conclusions

This study highlights the significant role that muscle strength improvements, particularly in the cervical region, play in the success of multimodal pain therapy for patients with cervical radiculopathy. The strength gains observed in this study serve as an objectively measurable indicator of therapeutic success, complementing the subjective reduction in pain. Specifically, cervical reclination strength increased by 22.9%, cervical inclination by 18.3%, and right and left rotation by 21.8% and 18.4%, respectively, which strongly correlated with a 54.2% reduction in pain scores on the Numeric Rating Scale (NRS). These results suggest that the targeted increase in muscle strength, especially in the neck, may directly contribute to the observed reduction in pain and improvement in function.

Additionally, while the peripheral muscle groups demonstrated smaller, non-significant improvements in strength, the primary gains in neck muscle strength underscore the importance of focusing on these key areas during treatment. This further supports the hypothesis that increasing strength not only in the directly affected regions but also in neighboring muscle groups can enhance the overall success of the therapy. While this analysis focused on clinically relevant movements such as inclination, reclination, and rotation, future studies could include lateral flexion to provide a more comprehensive assessment of the therapeutic effects.

The high interrater reliability (ICC of 0.98 before treatment and 0.97 after treatment) supports the robustness of the muscle strength measurements, but future studies with randomized designs and control groups are needed to confirm these results and explore long-term outcomes.

## Figures and Tables

**Table 1:** Example schedule of medical treatments for a 10-day multimodal pain therapy program.

Medical Application Within MPM	Number of Treatments
Medical training therapy	5
Group exercises	4
Aqua gymnastics	5
Progressive muscle relaxation	3
Coordination training group	4
Psychological group therapy/individual therapy	3

**Table 2:** Physical characteristics and pain scale NRS (Numeric Rating Scale, 0–10) of the patient group (mean and range values).

	All Participants (N = 25)	Women (N = 14)	Men (N = 11)
Age (years)	59.64 (42–84)	58.78 (42–77)	60.73 (42–84)
Height (centimeters)	171.72 (150–192)	164.5 (152–183)	180.91 (162–192)
Weight (kilogram)	81.02 (50–115)	76.14 (50–150)	87.23 (70–105)
BMI	27.4 (20.2–42)	27.99 (20.2–42)	26.4 (23.6–34.6)
NRS (on admission)	5.92 (2–9)	5.64 (2–9)	6.27 (3–8)
NRS (on discharge)	2.68 (0–9)	2.93 (0–9)	2.36 (0–7)

**Table 3:** Comparison of muscle group strength before and after treatment.

Muscle Group	Before Treatment (kg)	After Treatment (kg)	*p*-Value
Overall Strength	114.78	127.41	0.003
Cervical Inclination	10.79	12.76	0.005
Cervical Reclination	10.50	12.90	<0.001
Cervical Rotation Left	9.80	11.50	<0.001
Cervical Rotation Right	10.10	12.30	<0.001
Left Elbow Flexion	20.50	21.50	0.379
Right Elbow Flexion	20.00	21.40	0.153
Left Elbow Extension	17.20	18.00	0.198
Right Elbow Extension	16.00	16.90	0.072

## Data Availability

Data are available upon reasonable request.
